# Pathophysiological Link between Insulin Resistance and Adrenal Incidentalomas

**DOI:** 10.3390/ijms23084340

**Published:** 2022-04-14

**Authors:** Jordan A. Higgs, Alyssa P. Quinn, Kevin D. Seely, Zeke Richards, Shad P. Mortensen, Cody S. Crandall, Amanda E. Brooks

**Affiliations:** 1 College of Osteopathic Medicine, Rocky Vista University, Ivins, UT 84738, USA; jordan.higgs@rvu.edu (J.A.H.); alyssa.palmer@rvu.edu (A.P.Q.); zeke.richards@rvu.edu (Z.R.); shad.mortensen@rvu.edu (S.P.M.); cody.crandall@rvu.edu (C.S.C.); 2 Department of Research and Scholarly Activity, Rocky Vista University, Ivins, UT 84738, USA; abrooks@rvu.edu

**Keywords:** adrenal incidentaloma, insulin resistance, autonomous cortisol secretion, differential diagnosis of adrenal masses, subclinical hypercortisolism, adrenalectomy, pancreatic beta-cells, diabetes mellitus, metabolic syndrome

## Abstract

Adrenal incidentalomas are incidentally discovered adrenal masses greater than one centimeter in diameter. An association between insulin resistance and adrenal incidentalomas has been established. However, the pathophysiological link between these two conditions remains incompletely characterized. This review examines the literature on the interrelationship between insulin resistance and adrenal masses, their subtypes, and related pathophysiology. Some studies show that functional and non-functional adrenal masses elicit systemic insulin resistance, whereas others conclude the inverse. Insulin resistance, hyperinsulinemia, and the anabolic effects on adrenal gland tissue, which have insulin and insulin-like growth factor-1 receptors, offer possible pathophysiological links. Conversely, autonomous adrenal cortisol secretion generates visceral fat accumulation and insulin resistance. Further investigation into the mechanisms and timing of these two pathologies as they relate to one another is needed and could be valuable in the prevention, detection, and treatment of both conditions.

## 1. Introduction

**Figure 1:**
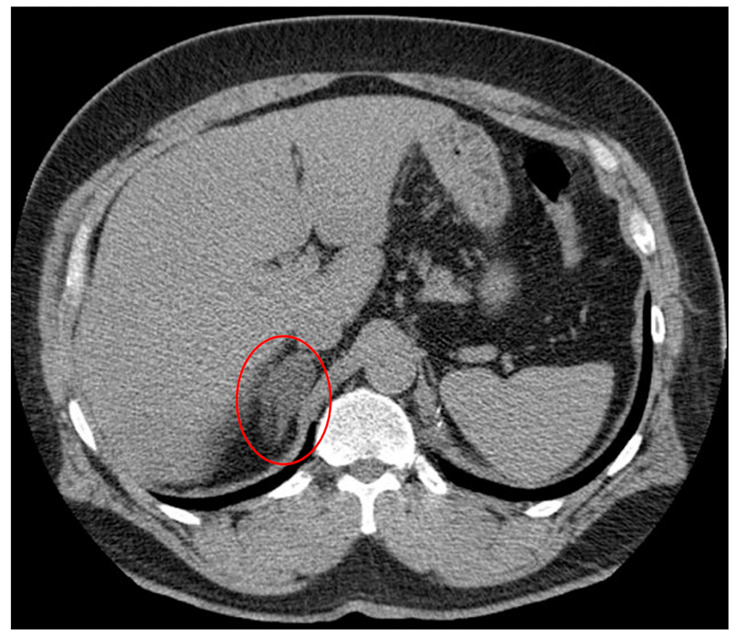
Incidental right adrenal nodule discovered on non-contrast CT. Case courtesy of Dr. Hani Makky Al Salam, radiopaedia.org. rID:10109 [9].

An adrenal incidentaloma (AI) is an adrenal mass found incidentally using imaging, independent of an endocrinological investigation (Figure 1) [1]. Technological advances and clinical integration of advanced imaging techniques, such as computed tomography (CT) and magnetic resonance imaging (MRI), for routine, often preventative, care has resulted in the increased detection of AI [2]. Independent of both the increasingly common use of imaging for routine healthcare as well as advances in imaging, the frequency of adrenal masses found incidentally via radiology is also increasing, with the current prevalence being 3–5 percent in imaging studies [3,4], compared with 0.5–2 percent in the 1980s and 1990s [3]. The increasing prevalence of AIs may reflect rising rates of insulin resistance (IR) [5], obesity [6], and hypertension [7] over the last twenty years [8]. The association between insulin resistance and adrenal mass formation is not well-characterized, although the link is concrete.

**Figure 2:**
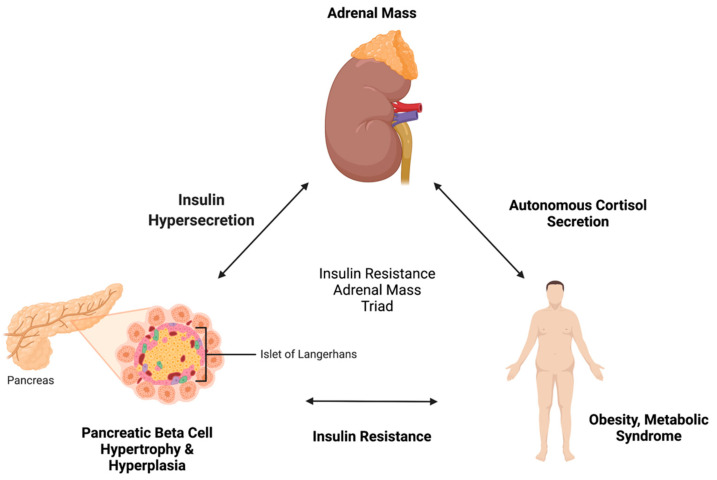
Obesity, metabolic syndrome, and resultant insulin resistance cause reactive pancreatic islet cell hyperplasia, hypertrophy, and insulin hypersecretion. In turn, this process has effects on adrenal tissue, causing cellular change and autonomous cortisol secretion. Figure created using biorender.com.

Some studies suggest that adrenal tumors elicit systemic insulin resistance; whereas others indicate the opposite pathway—that autonomous adrenal cortisol secretion may generate insulin resistance and obesity. It is plausible that both are true in a reciprocal triad (Figure 2). This review examines the literature pertaining to the pathophysiological mechanisms determining the interrelationship between systemic insulin resistance and adrenal masses, including functional, non-functional, benign, and malignant masses. First, background literature on the differential diagnosis of adrenal incidentalomas, the most common subtypes, and the production of autonomous cortisol secretion (ACS) with its associated downstream effects is reviewed to provide context to the discussion (Section 2). Next, the literature on insulin resistance is outlined as it relates to hyperinsulinemia and its effects on the adrenal glands (Section 3). The key relationships between adrenal hypertrophy, ACS, and insulin resistance, including genetic alterations, signaling pathways, and reciprocal interactions, are summarized and novel connections are posited (Section 4). Knowledge gaps and future research recommendations are discussed in Section 5, prior to a brief synthesis and conclusion (Section 6). In this narrative review, we aim to concisely characterize the complex and multi-elemental “adrenal-insulin axis” and identify critical gaps in knowledge and potential areas for therapeutic or preventative intervention.

There are key overlapping downstream effects of cortisol secreting adrenal incidentalomas that are hypothesized to underlie the reciprocal relationship between insulin resistance and adrenal incidentaloma. Downstream effects of benign functioning adrenal masses include subclinical hypercortisolism [10], Cushing’s syndrome [11] hyperaldosteronism [11], hyperandrogenism [12], local mass effects [13], and potential for malignancy [14]. Further removed downstream effects of hypercortisolism, potentially resulting from an adrenal incidentaloma, include obesity [15], weight loss [16], lipolysis [17], skin changes [18], muscle weakness [19], fatigue [20], hypertension [21], high blood glucose and sequelae [22], osteoporosis [23], menstrual irregularities [24], and insulin resistance [25]. Importantly, insulin resistance itself has a host of detrimental effects on the conditions of the body’s metabolic system, including reflex hyperinsulinemia [26], type 2 diabetes [27], high blood glucose and sequelae [28], atherosclerosis [29], metabolic syndrome [30], cardiovascular disease [31], and lipolysis [32]. The pancreas responds to insulin resistance and resultant high blood glucose by hypersecreting insulin, an anabolic endogenous hormone, which can elicit changes in insulin-like growth factor-1 receptor crosstalk [33], organomegaly [34], hepatic growth hormone receptor activity [35], TNF-α production by adipose tissue [36], insulin-induced-insulin-resistance [37], and adrenocortical tumors (Figure 2) [5].

Methods: Initially, major medical databases including PUBMED and MEDLINE were queried using the search terms adrenal incidentaloma and insulin resistance; the search was limited to developments from the past five to ten years. Subsequent in-depth, searches as topics and relationships were uncovered included topics specific to the pathology, radiology, etiology, and symptomatology of adrenal incidentalomas and insulin resistance as well as associated key pathophysiological interactions. Original research articles published radiological and clinical research studies, and literature reviews were all included in the current study. Key knowledge gaps in the literature were identified and evidence-based recommendations were formulated based on the collective expertise of the authorship and medical literature. Original figures were created using the Biorender platform.

## 2. Adrenal Incidentaloma

Adrenal incidentalomas (AIs) are masses of the adrenal gland measuring greater than one cm in diameter found unexpectedly on imaging performed for indications other than evaluation of adrenal pathology [38]. The detection of AIs has risen markedly in recent decades due in part to the increased utilization of high-resolution imaging techniques such as CT, MRI, and ultrasound [1]. AIs comprise some of the most common unexpected findings on imaging, seen on approximately 4% of all abdominal CT scans [3,4] (example in Figure 1). Older age is associated with an increased prevalence of AIs, rising up to 7% in patients over the age of 70. However, it is uncommon to discover AIs in patients under the age of 40. If found in patients less than 30 years old, timely evaluation for adrenocortical carcinoma (ACC) or functional masses is indicated [1].

**Figure 3:**
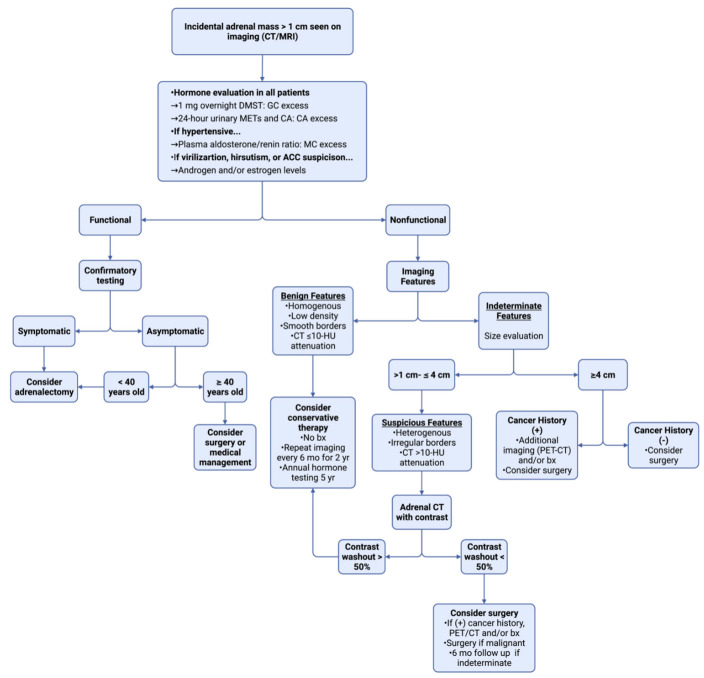
An organized approach to initial AI management [45,46,47,48,49]. DMST = dexamethasone suppression test; GC = glucocorticoids; METs = metanephrines; CA = catecholamines; MC = mineralocorticoids; ACC = adrenocortical carcinoma; HU = Hounsfield units; bx = biopsy; + = positive; − = negative. Figure created using biorender.com.

Although some studies have reported AIs to occur more frequently in female patients [39,40,41], data regarding whether biological sex affects AI prevalence is still inconclusive [42]. Recent studies have reported a higher prevalence of masses in the left adrenal gland versus the right adrenal gland [42]. In a 2018 study of adrenal adenoma laterality, Hao et al. reported a higher prevalence of left-sided lesions versus right-sided lesions when masses were greater than or equal to 3 cm. However, the authors concluded their findings to be a result of detection bias attributed to either small (≤3 cm) right-sided lesions or missed bilateral involvement [43]. An important lesson to take away from these and other studies surrounding controversial risk factors is that imaging techniques alone cannot be used to reliably determine tumor functionality [1]. Due to the possibility of hormone secretion and/or malignancy, key diagnostic procedures must be utilized in order to assess the need for surgery and initiate appropriate interventions [44]. A systematic approach to the initial management of AIs is shown in Figure 3.

By definition, incidentalomas are discovered in asymptomatic patients or patients being evaluated for an unrelated condition. Potential etiologies of AI include but are not limited to adrenocortical adenoma, adrenocortical carcinoma, pheochromocytoma, and metastatic cancer [1]. Most AIs occur in the adrenal cortex as adrenocortical adenomas and can be classified as either non-functional or functional (hormone-producing versus not hormone-producing, respectively) [50]. While the majority of AIs are non-functional, benign lesions (80–90%), up to 20% of incidentalomas are classified as functional and secrete subclinical levels of excess hormone, most commonly cortisol [51]. In those adrenal masses that are found to be benign, 10–20 percent of patients have detectable autonomous secretion of adrenal hormones, most commonly presenting as autonomous cortisol secretion (ACS) [52].

ACS typically presents as subclinical sequelae of hypercortisolism [53] which may include regulatory abnormalities in the hypothalamic-pituitary-adrenal axis, hypertension [54] cardiovascular risk factors [55], obesity and metabolic syndrome [56], insulin resistance [57], type 2 diabetes mellitus [58], and increased mortality (Section 4) [59]. Patients with AI may experience vague symptoms of local compression as a result of large mass size or signs of excess hormone production by a functional tumor. Mass effects may manifest as flank pain or abdominal pain and should increase the suspicion for malignancy [1]. The likelihood of an incidental adrenal lesion being malignant is most strongly determined by the presence or absence of malignancy elsewhere. In cases where malignancy is present, up to 27% of incidental adrenal lesions represent adrenal metastases [60]. Although the incidence of primary adrenal malignancy is low, these lesions rapidly proliferate and have a poor prognosis, necessitating thorough evaluation and swift intervention [45]. The differential diagnosis for an adrenal mass and possible clinical symptoms of both benign and malignant lesions are listed in Table 1.

The clinical signs and symptoms of functional adrenal mass differ depending on the hormone being secreted in excess [1]. Subclinical ACS, or subclinical hypercortisolism (SH), is the most commonly encountered form of hormone overproduction in AI patients [61]. Although SH lacks the obvious characteristics of Cushing’s syndrome, it has been associated with an increased risk of comorbid cardiovascular disease (i.e., hypertension and dyslipidemia), osteoporosis, and metabolic disease (i.e., insulin resistance, Type II DM) [62]. Early AI identification and intervention, if indicated, are integral in the prevention of long-term, comorbid disease development [45].

**Table 1:** Differential diagnosis for adrenal mass—benign masses.

Type of Benign Mass	Possible Clinical Presentation	References
Adrenocortical Adenoma (~80%)		[1,2,44]
Non-functional (~75%)	Asymptomatic, discovered on imaging	[1,2,44]
Cortisol-Producing (~12%)	Muscle weakness, easy bleeding/bruising, obesity, flushing, CV events, osteoporosis; overt Cushing’s syndrome	[1,2,44]
Aldosterone-Producing (~2.5%)	Muscle cramping/weakness, hypertension, headache, fatigue, polydipsia, polyuria, osteoporosis	[1,2,44]
Androgen-Producing (~2.5%)	Feminization, virilization (i.e., excessive facial hair, acne, clitoromegaly, male pattern baldness, deepened voice), hirsutism	[1,2,44]
Estrogen-Producing (rare)	Men: decreased libido, testicular atrophy, gynecomastiaWomen: IUB ^1^, breast tenderness	[1,44]
Pheochromocytoma (~7%)	Paroxysmal headaches, hypertension, weight loss, sweating, palpitations, anxiety, hot flashes (50%)	[1,2,44]
Myelolipoma (rare)	Possible flank/abdominal pain, shock due to rupture/hemorrhage	[44,49]
Adrenal Cyst (rare)	Acute abdominal pain	[44,63]
Schwannoma (rare)	Compressive symptoms/abdominal discomfort with increased size	[44,64]
Ganglioneuroma (rare)	Primarily asymptomatic, even if large	[44,65]
Hematoma/Hemorrhage (rare)	Asymptomatic—history of trauma, stress, sepsis, surgery, pregnancySymptomatic—nausea, abdominal pain, fever, hypotension, vomiting	[44,66]
**Malignancy**		
Adrenocortical Carcinoma (~8%)	Compressive symptoms (abdominal and/or flank pain) in 30%, symptoms of GC ^2^, MC ^3^, or androgen excess, if functional—40–60%	[1,2,44]
Metastatic Cancer (~5%)	Weight loss, vomiting, history of smoking or cancer (primarily lung, then GI, kidney, breast); symptoms of adrenal insufficiency if bilateral (i.e., postural hypotension, hyponatremia, hyperkalemia)	[1,44]
Adrenal Lymphoma	Abdominal pain, B symptoms (fever, night sweats, weight loss)	[1,67]

^1^ IUB = irregular uterine bleeding, ^2^ GC = glucocorticoid, ^3^ MC = mineralocorticoid.

## 3. Insulin Resistance

Conceptualizing the relationship between adrenal incidentalomas and insulin resistance requires an appreciation of the role of insulin. Insulin is an endogenous anabolic peptide hormone secreted by pancreatic beta-cells and does not require a transport protein to travel throughout the body, where it can act on various organs through the insulin receptor [68]. The insulin receptor at the cell membranes of the liver, adipose tissue, and muscle is a transmembrane tyrosine kinase dimer. The binding of insulin to the tyrosine kinase receptor leads to its phosphorylation. Once the tyrosine kinase is phosphorylated, cytoplasmic insulin receptor substrate (IRS) is activated, which allows for the subsequent intracellular second messenger cascade [69]. The second messengers produced ultimately result in GLUT4 receptor translocation to the cell membrane and glucose uptake into the cell [70]. Therefore, insulin is directly responsible for cellular glucose uptake through the action of glucose transporters (GLUT) [71]. 

Insulin resistance is defined as a decreased response to a given amount of insulin and is a central contributing factor to the pathogenesis of type 2 diabetes mellitus (T2DM) [72]. The impaired response to insulin leads to decreased glucose in tissues and the subsequent development of hyperglycemia. Chronic sustained hyperglycemia can progress to dyslipidemia, hypertension, and type 2 diabetes mellitus [73]. Diabetes mellitus (DM) is a major cause of morbidity affecting an estimated 8.7% of the US population [74]. DM is the most common metabolic disease in humans, with the hallmark pathology hyperglycemia being mediated by insulin resistance [75]. Risk factors for diabetes include obesity, inactivity, and family history [76]. Beyond T2DM, systemic effects of insulin resistance include macrovascular complications such as increased cardiovascular disease, stroke, and peripheral arterial disease [28]. 

Although the exact mechanism of insulin resistance (IR) is not completely understood, adipose tissue is known to play a central role in the desensitization of this critical hormone [73]. Adipose tissue is a dynamic tissue that demonstrates metabolic activity and hormone production and secretion [77,78]. Elevated fatty acids in the blood can mediate a decrease in the function of the downstream signaling pathway of insulin through the inhibition of insulin receptor substrate (IRS)-1 and 2 [79]. The effect of elevated free fatty acids in the blood will be further discussed below in Section 4.2. Elevated levels of TNF-α in adipose tissue have been shown to impair the insulin signaling pathway in hepatocytes and adipose tissue [80]. 

One study showed that mRNA expression levels of TNF-α in adipose tissue in obese individuals are strongly implicated in the pathogenesis of insulin resistance through impairment of insulin signaling in hepatocytes and adipose tissue [80]. In murine studies, chronic treatment with TNF-α decreased insulin-stimulated glucose uptake in rat skeletal muscle, while targeted deletion of TNF-α or its receptors increased insulin sensitivity and glucose tolerance in obese rodents [81]. In response to increasing IR, the beta-cells in the pancreatic islets of Langerhans undergo hypertrophy and hypersecrete insulin to balance the body’s response to insulin. However, an overabundance of insulin in the body has undesirable anabolic effects elsewhere, including the adrenal glands. 

## 4. Pathophysiological Link between Insulin Resistance and Adrenal Incidentaloma

### 4.1. Insulin Resistance to Adrenal Incidentaloma

As a result of the cell’s inability to respond to normal insulin levels in the blood (insulin resistance), the human body raises the level of insulin in the blood to compensate for the insensitivity. Hyperinsulinemia is a term for chronically high levels of circulating insulin, which is typically linked to obesity and T2DM. Because insulin is recognized for its growth capabilities, it’s possible that a state of excess insulin, such as hyperinsulinemia caused by insulin resistance, could result in the birthing of diverse masses throughout the body [74,75,82,83,84]. Although the majority of the research that would support this supposition has been performed on tissues other than adrenal tissue, the pathways presented provide a plausible means whereby insulin resistance may cause or play a role in the growth of adrenal incidentaloma.

Insulin plays a major role in the activation of different receptors in the adrenal gland that lead to growth and development [85,86]. Additionally, in the adult adrenal gland, IGF-1Rs and M6P/IGF-2Rs are normally expressed in the adrenal cortex [87,88]. The occurrence of most AIs in the adrenal cortex would support the involvement of this pathway. IGF-1Rs and M6P/IGF-2Rs have also been found in different locations within the adrenal gland, namely, IGF-1R in the zona reticularis and zona glomerulosa. When stimulated, these receptors can lead to increased steroidogenesis and adrenocortical cell proliferation. Specifically, elevated IGF-2 levels and IGF-1R overexpression has been implicated as a common occurrence related to adrenocortical tumors [89,90]. While this mechanism may be a contributing factor to the formation of an adrenal incidentaloma, it is not the only known contributor to adrenal gland tumor growth. Mouse studies assessing the presence of IGF-2 overexpressing altered states and their relationship with adrenocortical tumors found that although IGF-2 overexpression is a contributor to increased risk of tumorigenesis in the adrenal gland, although it is not solely predictive of mortality or tumor development [91,92]. 

Another plausible mechanism that may lead to an adrenal incidentaloma is the upregulation of hepatic growth hormone receptors by hyperinsulinemia. Leung et al. [35] elucidated that the “dominant restraining effect on GHR (growth hormone receptor) translocation may be a mechanism of limiting hepatic GH action in the presence of hyperinsulinemia’’. In turn, increased IGF-1 stimulation would increase, which has been linked to cell proliferation. This connection seen between growth hormones and insulin may be a biochemical feedback loop in which lack of GHR leads to hyperinsulinemia and subsequent upregulation of GHR in the liver and expression of IGF-1 [93,94], leading to the hypothesis that a metabolic condition inducing hyperinsulinemia may be a source of increased activity of growth hormone and subsequent IGF-1 action leading to cell proliferation in the body. 

Cell growth and mitogenic effects that may lead to cancer have also been shown to come from insulin receptors (IRs) [95]. Previous research determined that the binding of IGF-2 to insulin receptors was of a very low affinity [96,97]. However, newer studies investigated the separate affinities of the two insulin receptor types. Insulin receptors are divided into two isoforms: insulin receptor-A (IR-A), which has a higher mitogenic effect and is found largely in fetal and cancer cells, and insulin receptor-B (IR-B), which has mostly metabolic effects and is found mostly in insulin target tissues (liver, muscle, and fat) [98]. Frasca et al. [99] found that IR-A binds both insulin and IGF-2. The binding of IR-A to IGF-2 was seen to be of an affinity similar to that of insulin. While activation of IR-A by insulin will lead to metabolic actions, the binding of IR-A by IGF-2 was shown to promote mitogenic action. If this unusual binding of IGF-2 to IR-A has been linked to mitogenic action, it may contribute to the process of adrenal incidentaloma growth and proliferation. These results were validated in later studies on the IR-A and IR-B receptors studied in the context of cancer [100,101,102,103]. 

Insulin’s effect on the liver’s production of insulin-like growth factor binding proteins (IGFBPs) is another pathway that has been reported as a route for cellular proliferation. Brismar et al. [104] studied seven insulin-dependent diabetic patients in whom they monitored in a fasted, insulin-withheld state for a twelve-hour period overnight. Blood was then sampled before and during insulin infusions over a three-hour period. They found that the fasting IGFBP-1 concentration levels were inversely correlated with insulin levels. Insulin infusion resulted in inhibited splanchnic IGFBP-1 production and a significantly increased quantity of IGF-1 within 120 min. They concluded that during a state of insulinopenia, levels of IGFBP-1 become elevated significantly, and they are decreased following insulin infusion resulting in elevated IGF-1. Thus, in a state of hyperinsulinemia, IGF-1 levels may significantly rise and induce mitogenic effects in the body [105,106,107]. 

Insulin also interacts with hyaluronan, which leads to tumor progression. Hyaluronan is an essential polysaccharide component of the extracellular matrix. It performs essential functions that help organize tissue architecture and additionally regulates cellular proliferation and migration. It does so by interacting with cell-surface receptors and by adhering to other molecules [108]. Twarock et al. [109] found that hyaluronan synthesis in the cell is dependent on elevated glucose concentrations. They performed experiments analyzing the hyaluronan-mediated proliferation, invasion, and metastatic potential using OSC1 cells responding to elevated glucose, decreased insulin, and inhibited glycolytic action. In addition to a strong mitogenic effect from insulin action on the cells, they found elevated proliferation accompanying the higher glucose supply. In this manner, a hyperinsulinemic state induced by insulin resistance may lead to increased expression of hyaluronan and hence increased cellular proliferation. Whether this mechanism of cellular growth may trigger the formation of an adrenal incidentaloma is unknown. However, it may be an additive factor that promotes the growth of adrenal masses.

### 4.2. Adrenal Incidentaloma to Insulin Resistance 

**Figure 4:**
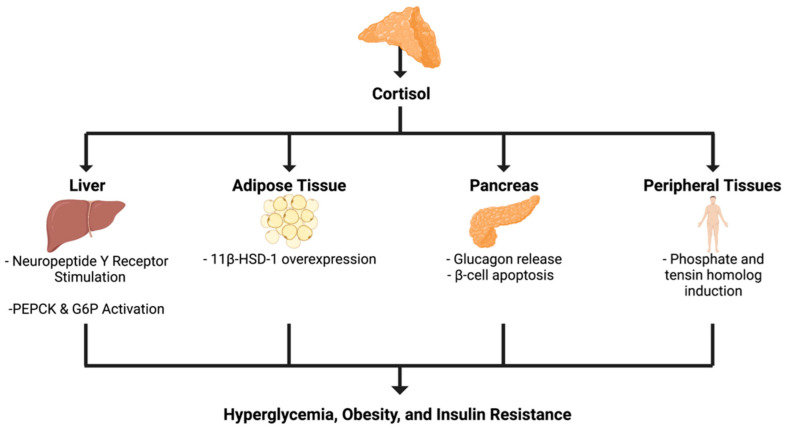
Direct effects of cortisol on body tissues lead to insulin resistance. Figure created using biorender.com.

Cortisol is normally secreted in a diurnal manner [110] and is under rigorous control by the hypothalamic-pituitary-adrenal axis [111]. Any deviation from the diurnal secretion or normal cortisol levels, such as in the case of ACS, has negative downstream direct and indirect effects (Figure 4). Chronic glucocorticoid (GC) exposure in humans is well known to result in whole-body insulin resistance and obesity. Numerous studies suggest the existence of elevated risk for developing insulin resistance, with ACS inducing greater risk compared to non-functioning adrenal masses [112]. In two open-label pilot studies [57,113] conducted in six and eight patients treated with the glucocorticoid receptor antagonist mifepristone for four weeks and up to three months, respectively, significant reductions in insulin resistance indices were observed in 5/6 and 6/8 patients studied. Furthermore, Androulakis et al. [114] showed that ACS patients without hypertension, diabetes, and with or without dyslipidemia exhibited increased IR and endothelial dysfunction as compared to patients with non-functioning adrenal masses. A causal or associative relationship in the pathophysiology of adrenal mass to insulin resistance may prove critical in this context. 

Cortisol stimulates hepatic gluconeogenesis through enzyme expression [115]. Under normal conditions, the main effect of glucocorticoids on glucose homeostasis is to maintain plasma glucose for the brain during stressful situations, as transiently raising blood glucose is necessary for optimal brain function. However, with constant glucocorticoid receptor (GR) stimulation at higher-than-normal levels, the effects are metabolically harmful. In a study by Asensio et al. [116], rats were infused with dexamethasone given intracerebroventricularly for three days, after which all rats developed hyperphagia, hyperinsulinemia, and insulin resistance. Hyperphagia, hyperinsulinemia, and insulin resistance were also observed after neuropeptide Y (NY) administration, leading the authors to hypothesize that the metabolic effects of glucocorticoid excess are mediated through NY receptor stimulation in the arcuate nucleus. Glucocorticoids also induce phosphoenolpyruvate carboxykinase (PEPCK) and glucose-6-phosphatase [117], further stimulating hepatic gluconeogenesis. Improvements in hepatic steatosis and hepatic triglyceride concentrations in fatty liver disease were demonstrated in a mouse model after hepatic glucocorticoid receptor disruption [118]. 

Glucocorticoid action is known to involve pre-receptor metabolism by enzymes such as 11β-Hydroxysteroid Dehydrogenase Type 1 (11β-HSD-1) that converts inactive glucocorticoids to their active form. Mice overexpressing 11β-HSD-1 in adipose tissue were shown to be obese and insulin-resistant [119]. Asensio et al. observed that adipose tissue 11β-HSD-1 microRNA expression is increased at the onset of high-fat diet-induced obesity and positively correlated with the degree of hyperglycemia. It is reasonable to assume this set of downstream effects would occur in the case of ACS and subclinical hypercortisolism as a result of a functional adrenal mass. A high cortisol environment impairs the insulin receptor signaling pathway in peripheral tissues through kinase activation [86]. In a study on the effects of cortisol on glucose uptake and the insulin signaling pathway in primary cultured endometrial epithelial cells, Qi et al. [120] showed that cortisol inhibited insulin-stimulated glucose uptake by induction of phosphatase and tensin homolog deleted on chromosome ten (PTEN) causing inhibition of Akt phosphorylation and glucose transporter type 4 translocation. 

In addition to stimulating its own production and activation, cortisol release from the adrenal glands acts directly on the pancreas to decrease the release of insulin from beta-cells while simultaneously acting on α-cells to stimulate the release of glucagon [121]. Glucagon acts by inducing glycogenolysis, liver gluconeogenesis, and lipolysis [122]. The induction of lipolysis by cortisol stimulated glucagon release contributes to the adrenal-insulin axis effect of insulin receptor insensitivity. Recent studies have shown that cortisol excess has an inhibitory effect on beta-cell activity [123]. The mechanism remains ambiguous but might involve cortisol’s direct influence upon the expression of molecules essential for glucose sensing and metabolism, enhanced glucose cycling, down-regulated insulin gene transcription, hampered insulin exocytosis, amplified alpha-adrenergic signaling, or increased beta-cell apoptosis. One study suggests RNA repressor GAS5 LincRNA involvement [124]. A mouse study evaluating the effects of chronic dexamethasone treatment showed INS-1 cell apoptosis mediated by dephosphorylation of Akt, Bad, and GSK-3-beta [125]. Other reports suggest that increased glucose-stimulated insulin secretion after beta-cell exposure to glucocorticoid in vitro, wherein transgenic mice with enhanced corticosterone regeneration within their beta-cells present augmented secretory capacity of their islets. 

The indirect effects of hypercortisolism and its action on insulin resistance involve hyperlipidemia-induced cell stress [28]. Oxidative stress at the cell occurs in the presence of excess glucocorticoid-induced hyperlipidemia and hyperglycemia [126]. Hyperlipidemia and hyperglycemia cause oxidative stress, endoplasmic reticulum stress, and mitochondrial dysfunction by upregulating both Jun N-terminal Kinase (JNK) and Inhibitor of Nuclear Factor kB Kinase (IKK). JNK and IKK cause the release of cytokines, which activate further JNK/IKK enzymes. JNK and IKK inhibit the insulin receptor substrate (IRS)-1 and 2, resulting in decreased responsiveness to insulin by the tyrosine kinase receptor (Figure 4) [79]. Glucocorticoids, therefore, induce insulin resistance via increased lipolysis in fat cells and subsequent hyperlipidemia and hyperglycemia. 

Glucocorticoid-induced hyperlipidemia and intracellular oxidative stress are potentially augmented by a subsequent decrease in plasma levels of adiponectin. Adiponectin is an adipokine that has been proven to influence metabolism and inflammation [127]. Adiponectin has been found to increase insulin sensitivity in both humans and rodents. Other therapeutic effects include anti-inflammatory, anti-atherogenic, anti-apoptotic, and weight decrease [127,128]. It has been suggested that oxidative stress decreases plasma adiponectin levels [129]. In cultured adipocytes, reactive oxygen species (ROS) exposure has been shown to suppress adiponectin mRNA expression and secretion [129]. Low levels of adiponectin have been tied to metabolic syndrome and insulin resistance. Low adiponectin levels lead to increased insulin resistance due to decreased tissue fat oxidation via peroxisome proliferator-activated receptor (PPAR) α activation, which subsequently increases circulating fatty acid levels and intracellular triglycerides in muscle and liver [130,131]. In animal models, decreased adiponectin preceded the onset of insulin resistance and diabetes. Individuals with visceral obesity were observed to have lower levels of adiponectin. Plasma adiponectin levels are affected by multiple factors, including gender, age, and lifestyle [132]. In the context of incidentalomas and high levels of glucocorticoid exposure and oxidative stress, a subsequent decrease in adiponectin may be an additional contributor to insulin resistance. 

**Figure 5:**
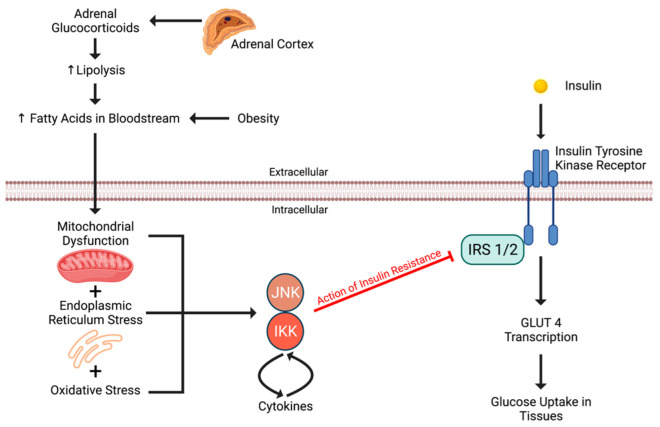
Adrenal glucocorticoids secreted from the zona fasciculata of the adrenal cortex induce lipolysis resulting in elevated fatty acids in the bloodstream. These fatty acids cause downstream inhibition of insulin receptor substrates 1 and 2 (IRS 1 and 2). This inhibition is critical in the development of insulin resistance. Figure created using biorender.com.

Glucocorticoid release from the adrenal cortex is seen in response to pathological processes that increase stress on the body, including malnutrition, infection, depression, and anxiety [133]. Increased glucocorticoid release is also seen as a symptom of adrenal masses [134]. Cortisol excess is also associated with pancreatic beta-cell dysfunction leading to impaired insulin secretion [123]. This impaired insulin secretion activates serine kinases to phosphorylate the serine sites on insulin receptors, which results in a decreased insulin signaling pathway activation by way of downregulation of tyrosine phosphorylation [135]. Lastly, glucocorticoids in excess increase hunger and decrease satiety by increasing leptin [136], leading to caloric excess, weight gain, and insulin resistance, as is the natural progression to T2DM (Figure 5).

## 5. Areas of Future Research

Further research is needed to elucidate the mechanism of action in both pathways. A significant majority of substantive original research took place in the 1980s and 1990s, with few original studies performed in the last 10 years despite the parallel rise in prevalence of both AI and IR. Importantly, the timing and onset of adrenal incidentaloma by subtype compared to the history of IR and IR onset would be valuable to better characterize this dynamic, multifaceted interrelationship. Investigation of the specific insulin receptors on adrenal cortical tissue and receptor variation in AI is needed, as are further studies into the mechanisms of excess glucocorticoid-mediated destruction or dysregulation of pancreatic beta-cells. These mechanisms may play a critical role in identifying and producing a correlative analysis of AI-predisposing and IR risk factors. Not all patients with IR develop AI, but nearly all patients with AI develop IR. Therefore, a Mendelian randomization process may be useful to investigate causative links between the pathologies.

## 6. Synthesis and Conclusions

There is significant clinical and basic biochemical evidence suggesting that AI can induce IR and that IR can promote AI. The direct and indirect effects of ACS on systemic insulin resistance are well characterized, including direct and indirect effects of cortisol on body tissues and pancreatic beta-cells. Cortisol-secreting adrenal masses may appropriately be included in differential diagnoses when considering clinical insulin resistance. The pathophysiological pathway of insulin resistance causing adrenal mass is less well characterized, although mechanisms for this pathway have been proposed, including the stimulation of IGF-1 and IGF-2 receptors. Furthermore, while the mechanisms of IR leading to increased mitotic activity and tumor proliferation are sufficiently established in the literature, causation has not been proven. Nevertheless, despite gaps in the underlying mechanism, there is sufficient data such that clinical insulin resistance and glucose monitoring should be considered when an incidental adrenal mass is discovered. After review of the literature, there is no currently accepted single thread to tie AI and IR together. The relationship can be characterized as reciprocal and self-enhancing in its progression with risk for severe disease if left to its natural course. Further investigation into the mechanisms and timing of the interrelationship is needed and could be valuable in the prevention, detection, and treatment of both conditions.
